# Distortions of the Feet

**Published:** 1874-11

**Authors:** 


### DISTORTIONS OF THE FEET.

We have lately given a good deal of our space to a discussion of this subject by Joel McComber, a practical Boot, Shoe and Last Maker for over thirty years, because we have long felt that the human race have never yet been properly dealt with in the matter of clothing for the feet. More than twenty-five years ago we wrote these words: "No other portion of the human frame is so tortured in the efforts made to clothe, protect and embellish it, as the feet. If the fashionable hat induced bunions, or if the close-fitting glove created corns, or if the cravat produced cripples, how quickly would the world be up in arms! Yet we go on from year to year, 'breaking in' our illy-formed boots and shoes, crippling and deforming the tender feet of our children, inducing enormous suffering, and laying the foundation of a multitude of diseases in the more vital organs, simply because shoemakers have no anatomical knowledge, and we have not the sense to appreciate the fact and to compel them to supply us with appropriate foot-gear." A quarter of a century has passed, and the facts are exactly the same to-day. The world has, meantime, learned a good deal about hygiene and diet and temperance, and a thousand subjects which make up the sum of human knowledge and contribute to human happiness. We have learned that our eyes and ears and teeth and digestion must have certain treatment and certain care, or they will fail us early; but we have not learned that thousands and thousands of men are actively employed day after day and year after year in constructing coverings for our feet which are certain to give us discomfort, certain to cripple us if our feet are not already distorted, and which will unfailingly keep up the distortion which barbaric foot-clothing has already secured.

We observe with sincere satisfaction that a step has at length been taken in the right direction. For the first time in the world's history, we believe, the true principle of constructing apparel for the feet has been discovered. That principle has been described in our pages by the discoverer, and we will not undertake to repeat it. But we will assert that we have satisfied ourselves that it is strictly correct, and that deformity of the feet is impossible, if the healthy, natural foot of childhood is clothed by this method, and the method is pursued through life.

Boots and shoes have been constructed, and are being constructed to-day, in all lands, with very small reference to the shape of the feet. There has been no real advance, no actual progress in this branch of science. As our grandfathers stood, so do we. If you will look at a last made one hundred years ago, you will find that it does not differ much in appearance, and not a particle in principle, from that upon which our Broadway shoemakers build their distorting foot-coverings. If you will look at any pair of boots or shoes in use, save only such as are made on the new last, you will notice one peculiarity: in all of them the upper leather extends beyond the sole on the outer side of the foot. In other words, the sole is not precisely fitted to the outline of the bearing surface of the foot. A portion of the upper leather thus practically acts as the sole. This is, of course, altogether wrong.

But there is a far worse error than this. If you will examine any last except McComber's, you will observe that the thickest part of it at the instep, and from the instep to the end of the toe, is in the centre. This is barbarity itself. Look from the abominable last in common use, to your foot. If your foot has not been distorted beyond recognition as a human foot, you will discover that the thickest part of your foot is along its inner side, from the instep to the extremity of the great toe. This will be fully apparent in the foot of the child who has never suffered from the forcing process which begins with the first little pair of shoes. Of course the first marked effect of wearing a shoe made on a last having its thickest portion in the middle, is to crowd the large toe over upon the other toes—to pile the toes up, in short, in a compact mass in the center, where the only space is. This deprives them of that elastic springiness of motion which attends graceful walking. It compels corns, also, and forces the sharp nails down into the surrounding flesh. But it does more and worse, far worse, than this. First, it tends to make children very backward in their efforts to walk. Their tender feet are compressed within leather sheaths so closely as to render it impossible for them to balance themselves. Again, the compression of the feet prevents the free circulation of the blood, and congestion of the vital organs is very liable to follow. There is no one cause more prolific of diseases to which the females of the human race are subject, than ill-constructed boots and shoes, and we intend to explain, some day, exactly why this is the fact.

What is the remedy for all this visitation of misery? Properly shaped boots and shoes, and of course properly shaped lasts to make them on. Remember, no "breaking in" process will accomplish the desired result. You may break in a mal-formed shoe to such an extent that you can manage to wear it, but if it is wrong in the outset, it will be wrong all through. The distortion continues, although the misery may be less apparent. Every step you take, forces your foot still further over the sole outwardly, dragging the upper leather with it and forcing the great toe still further out of a line with the rest of the foot, and so perpetuating the deformity. We have no doubt that club-foot and other pedal deformities have been occasioned by bad shoes, and we wish to impress upon parents the necessity of having the tender feet of children properly cared for. Mr. McComber is an enthusiast in his profession, an honest and worthy man, and we commend him and his grand improvement, with earnestness to the confidence of our readers. He may be found at No. 21½ Spruce street, New York, where he makes lasts to fit all feet, and first-class boots and shoes at once elegant, durable and comfortable, for all ages and both sexes. Persons need wear boots and shoes which deform their feet no longer; if they prefer to, however, let them not blame us.

